# Memorias Sobre La Topografia Medica De La Habana

**Published:** 1857-11

**Authors:** 


ART. IV.—*Memorias sobre la Topografia Medica de la Habana y sus Alrededores, y sobre el Estudio Fisico et Moral de los Colonos Asiaticos.* Por el DOCTOR DON MARCIAL DUPIERRIS, &c. &c. Habana. P—s 106. 8vo. 1857.

*Memoirs on the Medical Topography of Havana and its Environs, and on the Study, Morally and Physically, of the Asiatic Colonists, &c.* By DR. MARCIAL DUPIERRIS. Havana. 1857.

THESE memoirs, as we learn from the author's advertisement, form part of an unpublished work which he wrote in 1851, in compliance with an order from the Director of the Board of Health of the Royal Navy. Her Majesty the Queen, in consequence of the Report of the Royal Academy of Medicine and Surgery of Madrid, ordered its publication, the expense to be defrayed out of the funds granted for public instruction. But it is one thing to issue a royal decree at Madrid, especially where it involves an outlay of money, and another to carry it into effect in Havana; and, accordingly, we learn that the author, while enjoying the honor of the royal mandate, has deemed it advisable to print himself a portion of the larger work. Dr. Dupierris dedicates the present publication to the City of Havana, and the planters of the island of Cuba.

In his introduction, he adverts to the changes in the atmosphere as modifying the occult causes of disease,—malaria, for example, and the effluvia which contribute to the production of yellow fever. Arago and Schubler are mentioned as explaining the power of winds in transporting effluvia, by saying that this depends, in some measure, on the hygrometrical state of the atmosphere, and that the accumulation of electrical fluid at the surface of the earth, during moist states, favors, perhaps, the evolution of effluvia. After speaking of the infinite number of circumstances under which effluvia are generated, he instances a combination of elements for this end in the mixture of fresh and sea-water, as related by M. Melier, in the salt marshes of Lucca, on the Mediterranean coast. Periodical fevers, which had prevailed before, ceased to recur in this district when the water of the sea was prevented from flowing into the fresh-water marshes. Reference is made to the belief of M. Chevreul, and other writers, in the morbific agency of sulphuretted hydrogen gas. Whatever may be the elements which go to form effluvia, we cannot but consider their presence as injurious; and although we do not always perceive the deleterious operation of these latter, there is no reason, continues the author, why we should not take all pains to free ourselves from their presence.

The first chapter of the memoir, on the topography of Havana and its environs, opens with the assertion, that the study of topography is an indispensable preliminary for acquiring a knowledge of diseases: reference is made to the authority of Hippocrates to this effect. In Havana, as elsewhere, the cholera has proved a wonderful inciter to the study of meteorology and topography. It is in this connection that Dr. Dupierris exhibits the result of his observations on the existing causes of the unhealthiness of that city, and indicates the means by which it, the richest and fairest of the Antilles, may be made the most healthy of all of them.

"The City of St. Christopher of the Havana" is situated a few miles within the tropic of Cancer, or in latitude 23° 9′ north, and in longitude 76° 4′ from the meridian of Cadiz (or 82° 23′ from London). Its population, which, in 1823, was 112,033, is now 134,122, of which 74,623 are whites. It occupies an area of 300,000 yards (*varas*), the surface of which exhibits various degrees of undulation around the bay. The elevated part is spread fan-like, with its surface concave, and at its end, or obtuse angle thus formed, are several openings; some in the walls, which give on the street of Cuba; others into the sewers which carry off the waste waters into the harbor. In the Alamada of Columbus, in the quarters of Jesus Mary and of Carraguao, the soil is very marshy. The unhealthy space here is being diminished by draining, and the construction of handsome buildings. The streets run from north to south, and from east to west: they are rather narrow, and not paved, and are deficient in cleanliness. In some of the new suburbs the streets are wider and better laid out; but the sidewalks, naturally bad, are less attended to than in the city proper.

The houses of Havana are generally constructed on the same plan, each of them having a portico ten yards by twelve. On one side is the hall, which is connected with different rooms, opening one into the other. Fronting, there is a court, beyond which, and of not very inviting appearance, are the kitchen, stables, privy, lodgings of the coachman and other servants, sheds for horses-gear, and vehicles of different kinds, all in "most admired disorder." When the doors between the rooms are left open, a strong current of air is established, which often causes suppressed perspiration in persons thus exposed, followed by functional disorders, and even spasms, which are of such common occurrence in tropical regions.

In a great many houses in Havana, of less size and value than those just described, the portico is wanting, and the hall is then a general thoroughfare for persons and animals, the barrel of night-soil, all, in fine, that is collected in the rear sheds. The hall itself is damp and dirty. It is not difficult to anticipate the inconveniences, and even fatal consequences of such a state of things; but still more deplorable results must obtain from the emanations from the open sinks and drains, which are between the kitchen and stables, and which receive the waste waters and refuse from them, and sometimes the barrel of night-soil itself. This latter is generally carried away daily, at noon, in carts, by persons who contract to perform the work. The passage of these carts is, of course, accompanied by a horrible stench, which always announces their approach. Well may the author say that if Havana, in the midst of these elements of pestilence, should not be all the time scourged, it must be regarded as proof of the excellence of the climate itself. The kitchen has but one door, which opens on the court, and as the ground before is neither paved nor tiled, it is continually wet, if not muddy, by the water and slops thrown on it. The court in this class of houses is too small to allow of due access of air to the rooms which open into it, and which, as there is no second story, are those occupied by the family. They are also often shut out from the sun's rays, and adequate access of fresh air by lofty buildings near them. The privies want wells, and are in other respects badly constructed, there being no tube for carrying off fetid gaseous exhalations, nor other means of escape for these than the door: the floors are also incrusted with dirt.

Public hygiene would seem to be retrograding in Havana. For a while the fluid portion of the night-soil was put into well-closed vessels, and the solid part into others which were air-tight; but notwithstanding the obvious advantages of such an arrangement it was discontinued, because the contractors could not agree to perform their work on as low terms as others who followed the old and nasty fashion of carrying away all the contents of the privies together. Formerly, the carrying away these matters was done in the night instead of as now in midday. Another injury to public health consists in distributing the night-soil, without prior preparation or its being made into compost, on the fields in the neighborhood of the city. By this means, myriads of flies and other insects are attracted to the spot, and destroy the plants or other vegetable growth, the decay of which, with the death of these insects, contribute greatly to deteriorate the air of the spot. Some useful suggestions are offered by the author for the improvement of sewers, and the introduction into them of fresh water, and openings, at intervals, for ventilation.

He next describes the suburbs, and the infirmaries and asylums in these quarters. The public cemetery is here, as cemeteries have been so often elsewhere, a nuisance. The soil is sandy and only two or three feet deep, and rests on a hard rock. The emanations from the decomposition of bodies deposited here are sensibly felt, and but for the prevalence of winds from the east and east-northeast, which waft them in another direction from that of the inhabited part, the worst effects would result. The district of Jesus Mary, situated on the south side of the city, which latter, of course, receives the winds blowing west, is in peculiarly wretched condition on the score of hygiene. The streets are for the most part narrow, badly laid out, unpaved, and with the surface broken into inequalities, and obstructed with impurities. In other places, one is disgusted with the sight of filthy ponds or stagnant pools and marshes, the waters of which have no exit: ditches also apparently to receive rain water, the refuse and offal from houses, and a heterogeneous mixture of all kinds of abominations, among which are included dead animals and the like. With the exception of five or six streets in this quarter, all the others are impassable. The houses and inhabitants correspond with the streets; all showing filth and squalor. Another source of pestiferous exhalations in this quarter is the canal or drain of Chavez, which traverses the marshes of Penalver, whose waters it receives; its own holding in suspension the decomposed filth of the city: and at the Matadera it is the recipient of the blood, offals, and excrements of the cattle brought here for slaughter. Other nuisances, ponds, &c., are seen in this wretched quarter, the very name of which ought, one would suppose, to urge every devout Spaniard either to change its title, or, this remaining, to institute such hygienic improvements as are demanded. El Cerro, about a league from Havana, though its ground is low and humid, is the most fashionable quarter in the neighborhood of the city, and one prized for the freshness of its air and the baths of running water, which is introduced into the houses.

In that part of the harbor on which the arsenal is situated (*Puerto del*

*Arsenal*), have been sunk ships of the line, frigates, and smaller vessels, which are buried in the mud, and are only partially visible at low tide. Following the shore, we come, in a south-southwest direction, to the *puerto de Tallapiedra*. In this part is the Military Hospital, which, in common with the city itself, is exposed to winds from the south that blow over mangrove lagoons and the Canal of Chavez. The latter empties into the harbor in this direction, charged with all its putrescent abominations. The water of this part of the bay is nearly stagnant, and being scarcely changed by the tides, it contributes its full share to the pestiferous exhalations just mentioned. Continuing our course round the bay, we meet, at the distance of a mile from Fort Atares, with the Bay of Guasabacoa, and mangrove thickets and marshes. The ground is partly covered by the village of Regla, large public warehouses for preserves and comfits, and islets which are embosomed in it. This bay is, in the opinion of Dr. Dupierris, the most unhealthy part of the harbor of Havana. Its waters are stationary, and furnish ample material for the evolution of effluvia destructive to human life. A continuation of the circuit of the harbor exhibits to the observer fresh marshes and fresh sources of disease.

Havana is now supplied with water brought by the Aqueduct of Ferdinand VII. It first flows into large basins, where it is filtered, or rather loses by deposit its earthy and other matters suspended in it: thence it is distributed through the city in iron pipes. It is rather hard, and acts sometimes harshly on the digestive canal, so as to predispose to disease, in seasons of epidemic visitation, such as cholera.

The author terminates his memoir on the medical topography of Havana by a summary of the causes, which we have briefly noticed, of its unhealthiness, and the means for their abatement or removal. We will not follow him in his recommendations, which are apposite and useful; but as coming within the more generally known exactions of public hygiene, they need not engage our notice at this time. There is, assuredly, great call for an enlarged and systematic plan of amelioration and reform in all that relates to the public health in Havana.

Into a consideration of the subject of the second memoir of Dr. Dupierris, viz., on the physical and moral condition of the Asiatic colonists (coolies), we cannot enter at present. Involving, as the subject does, many points of hygiene, therapeutics, and political economy, it would require time and space, beyond our ability just now to command.

